# Melatonin as a Possible Natural Safener in Crops

**DOI:** 10.3390/plants11070890

**Published:** 2022-03-27

**Authors:** Manuela Giraldo Acosta, Antonio Cano, Josefa Hernández-Ruiz, Marino Bañón Arnao

**Affiliations:** Department of Plant Biology (Plant Physiology), Faculty of Biology, University of Murcia, 30100 Murcia, Spain; manuela.giraldoa@um.es (M.G.A.); aclario@um.es (A.C.); jhruiz@um.es (J.H.-R.)

**Keywords:** biostimulator, crop, fungicides, herbicides, insecticides, melatonin, pesticides, phytomelatonin, plant stress, safener

## Abstract

Melatonin is a well-known animal hormone with relevant and multiple cellular and hormonal roles. Its discovery in plants in 1995 has led to a great diversity of molecular and physiological studies that have been showing its multiple actions also in plants. Its roles as a biostimulator and modulator agent of responses to abiotic and biotic stresses have been widely studied. This review raises the possible use of melatonin as a natural safener in herbicide treatments. Existing studies have shown excellent co-acting qualities between both the following agents: herbicide and melatonin. The presence of melatonin reduces the damage caused by the herbicide in the crop and enhances the stress antioxidant response of plants. In this area, a similar role is suggested in the co-action between fungicides and melatonin, where a synergistic response has been demonstrated in some cases. The possible reduction in the fungicide doses is proposed as an eco-friendly advance in the use of these pesticides in certain crops. Finally, future research and applied actions of melatonin on these pest control agents are suggested.

## 1. Introduction

Safeners are chemical compounds used to reduce the harmful effects of herbicides on crop plants and are applied alongside the herbicide. Safeners, also known as herbicide antidotes, are usually presented in mixed formulations of herbicide and safener [1]. Generally, they are often used to control weeds in large crops such as grasses, corn, rice, etc. Safeners appeared as a new tool in 1971, with the first safener (1,8-naphthalic anhydride) launched by Gulf Oil Company under the trade name Protect for the treatment of maize seeds. This first safener was developed against pre-emergence thiocarbamate herbicides such as EPTC (S-ethyl dipropylthiocarbamate) [2]. Table 1 shows the most commonly used safeners in different crops. Most of the safeners were developed in the 80s and 90s, achieving a breakthrough with mixed formulations of herbicide and antidote since they facilitated their handling and application. A particular case is that of daimuron, cumyluron, and dimepiperate, which are actually registered as herbicides. However, all three have a protective effect on rice, especially against sulfonylurea herbicides, which was discovered fortuitously when different mixtures of safeners and sulfonylurea herbicides were developed. Because the safeners are usually incorporated into the herbicide, farmers do not usually give much importance to them since the unique objective is weed control. However, it is estimated that the market value of these safeners was around €1.7 billion in 2011 [1].

As for the way the safeners act, today we know that these compounds mainly act by inducing the degradation of the herbicide in the tissues of the crop plant. The active degradation of the herbicide to non-harmful compounds causes less cellular and physiological damage, so the safeners protect the plant from excessive exposure to the herbicide. The rate of herbicide degradation is intimately related to crop selectivity and weed control. Thus, if the rate of degradation to inactive forms of the herbicide in the crop is very slow, then damage will occur. On the other hand, if detoxification is too fast in weeds, then the weed control effect of the herbicide is reduced or lost. Therefore, with the added safener, the herbicide is metabolized more quickly and, as a result, the threshold that would cause visible damage to crops is not reached [1,3,4]. The degradation steps of the herbicides are known, which mainly go through activation reactions by cytochrome oxidase P450, conjugation reactions with glutathione, sugars, and/or amino acids, and finally, a step of translocation to the vacuole with the subsequent action of tonoplast transporters [5,6,7,8]. It is known that, in some cases, many of the enzyme genes involved in herbicide degradation reactions are upregulated by safeners. So that the safener alerts and prepares the plant cell for the subsequent reduction of herbicide levels, minimizing the toxic effects of this. The set involves the activation of the defense and detoxification genes well-known in situations of chemical stress, where it has also been proven that salicylic acid is induced by safeners and has a prominent role in this defense [9,10,11]. Therefore, it appears that several signaling pathways may contribute to the complex protective response in plants. However, the main purpose of the safener’s signaling is still unknown. Future studies may provide a clearer understanding of these signaling cascades and also help explain why protectors work well on specific crops and not on weeds [12,13].

In some cases, the safener has been proven to act as an activator or inactivator of the herbicide. Some safeners can perform the activation of the initial compound in the form of a pro-herbicide, generating the active form of the herbicide in the plant tissues (for example, the hydrolysis of fenoxaprop-P-ethyl inactive to its active form, fenoxaprop-P acid) [14]. A particular case is that of dietholate. It inhibits the appearance of the active herbicide from the pro-herbicide form. Specifically, the activated herbicide clomazone is generated by the action of cytochrome P450, which is inhibited by dietholate in plants, reducing crop damage [15]. Isoxadifen-ethyl, commercialized in 2002, was the first safener with strong multi-crop (corn and rice) and multi-herbicide post-emergence activities. One of the latest commercialized safeners was cyprosulfamide, launched in 2009. It is strongly active in corn and sorghum and is particularly interesting because it can protect against both pre- and post-emergence herbicides. However, no new safeners have been authorized in recent years due to restricted legislative limitations on the use of synthetic substances, which can present serious ecotoxicity problems. That is why there is a great opportunity to use natural safeners such as melatonin in crops.

In this review, we analyze the results published so far to be able to consider melatonin as a possible natural plant safener. The properties of melatonin as a biostimulating agent in stressful conditions and its possibilities as a safener against treatments with herbicides, fungicides, and other pesticides are presented. Its possible use in crops is proposed with important considerations in its application.

## 2. Melatonin in Plant Growth and Abiotic/Biotic Stress

Melatonin (*N*-acetyl-5-methoxytryptamine) was discovered in 1958 in the pineal gland of a cow by Lerner et al. and then in humans, elucidating its chemical structure [16,17,18]. Melatonin is an important hormone regulating day/night rhythms, acting as a chronobiological signal that provides information to the brain and peripheral organs [19]. In 1995, the unequivocal identification of melatonin in plants was made [20,21,22]. It took about 10 years to begin unraveling many of the functions that melatonin exerts on plants [23]. Actions such as germination, growth, and rooting of plants, and also as a leaf senescence retardant, were the first functions proposed for melatonin in plants [24,25,26,27]. Moreover, an extraordinary role as a protective molecule and an activator of tolerance against stressors were suggested in the first studies [28,29,30,31]. Currently, it has been shown that melatonin acts as a hormonal molecule with the capacity for biostimulation of plants in the face of abiotic stresses such as drought, waterlogging, salinity, cold/heat, toxic agents, heavy metals, UV radiation, etc. [32,33,34,35,36,37,38,39,40,41,42], and also against biotic stress caused by plant pathogens such as bacteria, fungi, and viruses [43,44,45,46].

## 3. Melatonin as a Plant Biostimulator

**Figure 1:**
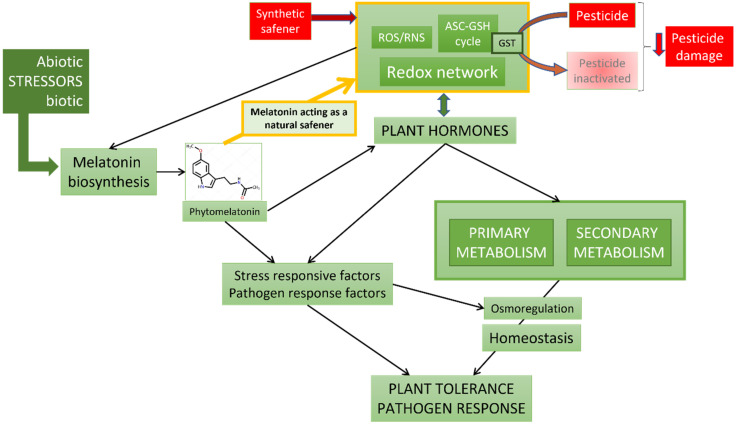
General model of melatonin action in abiotic and biotic stress responses according Arnao et al. [35,42]. The different elements integrated in the response of melatonin to stress situations are represented in green boxes, including the possible action as a natural safener against pesticides. Red boxes show the action of synthetic safeners and the activation of the pesticide detoxification mechanism through glutathione-S-transferase (GST), reducing the harmful effects of pesticides on the plants.

The antioxidant properties of melatonin have been widely studied and are well known. This indolamine has a high antioxidant capacity against reactive oxygen species (ROS) and also nitrogen (RNS), in vitro and at the cellular level [47,48,49,50,51,52]. Melatonin’s powerful antioxidant capacity (up to 4times greater than that of vitamin C and E) is usually accompanied by the expression induction of several genes related to the antioxidative defense response. In both animal and plant cells, the induction capacity of the elements of the ascorbate-glutathione (ASC-GSH) cycle has been demonstrated [42,53,54,55]. Thus, plants under stress conditions usually produce melatonin biosynthesis in their tissues, activating melatonin biosynthesis genes and subsequently activating the anti-stress response that involves the synthesis of antioxidant metabolites (mainly ascorbate) and ROS-eliminating enzymes such as catalases, peroxidases, glutathione reductases, glutathione transferases, etc. All this results in control of the redox network and a re-establishment of cellular homeostasis [35,56,57]. The action of melatonin in plants is much more complex and global. In stress conditions, melatonin increases the tolerance response through the control of key transcription factors, such as DREs (dehydration-responsive elements), CBFs (C-repeat-binding factors), MYBs (regulator of CBFs), WRKYs (transcription factors involved in biotic and abiotic stress responses), NACs (auxin-related factors), ERFs (ethylene-responsive elements), and the more specific ZATs (ROS-related responsive elements), cold-responsive genes such as COR (anti-freezing protein response), LTI (low-temperature induced factors), KIN (anti-freezing protein response), and RD (responsive factors to dehydration); HSFs (heat shock factors) in heat-stress, up-regulating several heat-sock proteins (HSPs); CBLs (calcineurin B-like proteins) and CIPKs (CBL-interacting protein kinases) in salt-stress, among others [58], regulating adequate responsive elements that imply the adjustment of several elements in primary and secondary metabolic pathways [42,59]. Possibly, many of the melatonin-mediated actions are executed through other well-known plant hormones such as auxins, gibberellins, cytokinins, abscisic acid, ethylene, jasmonic acid, salicylic acid, and others. Melatonin has the ability to regulate elements of biosynthesis, catabolism, and signaling of all plant hormones, so it has been called a plant master regulator [35,42,57,60] (Figure 1).

## 4. Melatonin as a Possible Natural Safener: Examples

The role of melatonin as a protector in biological systems has been widely demonstrated. In animal cells, melatonin reduces oxidative stress against drugs, toxins, and heavy metals [61]. Moreover, against highly toxic agents such as sulfur mustard, a chemical warfare compound, a protective effect of melatonin has been described [62,63]. High levels of malondialdehyde and 4-hydroxyalkenals (lipid peroxidation products) were reduced in the serum and lungs of rats with the co-administration of paraquat and melatonin (5 mg/kg) [64]. Similar results have been shown with diquat (a similar herbicide) in the liver and kidney of mice, which increased serum aminotransferase levels and reduced the acute 24-h death rate in melatonin/diquat-treated mice from 91% to 57% [65]. In an interesting study on honeybees, T5H-overexpression (tryptamine 5-hydroxylase, a rate-limiting enzyme of melatonin biosynthesis) in bees induced endogenous melatonin levels and oxidative stress tolerance against abiotic stressors such as low temperatures, UV exposure, and pesticide (paraquat, cyhalothrin, abamectin, and bifenthrin) treatments, increasing the survival rate [66].

Table 2 shows the different studies on pesticides together with melatonin as a possible safener in different plants. Several herbicides have been checked. The first tested was the uracil-type herbicide called butafenacil (1-(allyloxycarbonyl)-1-methylethyl-2-chloro-5-[1,2,3,6-tetrahydro-3-methyl-2,6-dioxo-4-(trifluoromethyl)pyrimidin-1-yl]benzoate), developed by Syngenta Co., Basel, Switzerland, in 2003.

Butafenacil is an herbicide used to control annual and perennial broad-leaved weeds that inhibits the protoporphyrinogen IX oxidase enzyme involved in chlorophyll biosynthesis, provoking a massive accumulation of chlorophyll precursors and generating dangerous singlet oxygen molecules and membrane lipid peroxidation, leading to cellular death [74]. In the presence of butafenacil, melatonin-rich transgenic rice plants that overproduce endogenous melatonin showed resistance to the herbicide, containing high chlorophyll levels and low malondialdehyde (MDA) and hydrogen peroxide content, and also high superoxide dismutase (SOD) and catalase activities compared with wildtype rice plants. This initial paper opened the door to the consideration of melatonin as a possible safener [67].

Paraquat, also dimethyl viologen (1,1’-dimethyl-4,4’-bipyridinium dichloride) is a broad-spectrum herbicide that blocks the process of photosynthesis at the photosystem I level, generating excessive ROS production with critical damage [75]. Pea plants with their seeds previously hydro-primed with melatonin and treated with paraquat showed high stability and improved functioning of photosynthetic pigments, enhancing their oxidative stress tolerance compared with untreated plants [68,76]. Poplar leaves pretreated with melatonin exhibited increased tolerance to paraquat-mediated oxidative stress. Melatonin reduced membrane damage and lipid oxidation in poplar leaf discs and also stimulated antioxidant enzyme activities such as SOD (superoxide dismutase), catalase, peroxidase, and ascorbate peroxidase, increasing antioxidant metabolites including ascorbate (ASC), glutathione (GSH), and proline in leaves exposed to paraquat. All this showed the ameliorative effect of melatonin on the damage caused by the herbicide in poplar [69].

A potential herbicide used in sweet potato (*Ipomoea batatas*) crops to control yellow nutsedge (*Cyperus esculentus*) weeds is bentazone (3-isopropyl-1H-2,1,3-benzothiadiazin-4(3H)-one 2,2-dioxide), a post-emergence contact diazinone herbicide (inhibits Photosystem II action at D1 protein level) used to control annual weeds in a variety of crops [77]. Bentazone in sweet potato seedlings caused severe injuries (41–75% losses, depending on variety tolerance), even at low doses (0.1 mM). The use of melatonin as a safener led to 30% less damage, doubling the biomass yield compared to treatments with the herbicide alone. The authors suggested using melatonin as a possible safener in weed control [70].

The term “safener” was first described in 1971 and applied to the protective effect against herbicides. However, we can make an extension of the safener term by covering other pesticides such as fungicides, insecticides, acaricides, and others. In the case of fungicides, melatonin has been used to minimize damage or even enhance fungicidal action. In tomato plants, the fungicide carbendazim (methyl benzimidazol-2-ylcarbamate), which acts by inhibiting fungal mitosis, also provokes pesticide-induced phytotoxicity, causing severe oxidative stress in treated plants. Co-treatments with exogenous melatonin alleviated ROS production and lipid peroxidation. Moreover, a modulation of the ASC-GSH cycle by melatonin occurs, increasing ASC, GSH, and antioxidative enzyme levels, improving the detoxification capacity of the plant cells and metabolizing fungicide with minimal collateral damage. According to the authors, melatonin makes plants “cleverer” to withstand phytotoxic stressful conditions [71].

Ethylicin (S-ethylethanethiosulfonate) is a biofungicide with broad spectrum activity, mainly used in oomycete disease control. In agriculture, pathogenic oomycetes such as *Phytophthora* sp. are one of the most devastating diseases. In a study of tobacco black shank (*P. nicotianae*) using ethylicin and melatonin co-treatments, both compounds induced the inhibition of the hyphal growth, the reduction of the cell viability, and the suppression of the virulence of *P. nicotianae.* Moreover, melatonin and ethylicin shared the same metabolic targets, interfering amino acid metabolism, overexpressing apoptosis-inducing factor, and dysregulating the virulence-related genes. The authors proposed that tobacco black shank caused by *P. nicotianae* can be successfully controlled using the combination of ethylicin and melatonin as an eco-friendly alternative for the control of the oomycetic diseases [72]. In a similar study of potato late blight caused by *P. infestans*, melatonin inhibited mycelial growth and increased stress tolerance, attenuating the potato late blight symptoms. In the use of the fungicide Infinito^®^ (a mixed combination of fluopicolide and propamocarb), synergistic antifungal effects of melatonin with fungicides were described, suggesting that melatonin could reduce the dose of fungicide and improve the efficacy of the fungicide against late potato blight [73].

A general scheme is proposed (Figure 1), where the known role of melatonin in situations of abiotic and biotic stress, is added to the role as a possible natural safener. In the case of artificial/synthetic safeners, their action as modulators of the redox network is proposed, in addition to their well-known detoxification/inactivation at the level of the ASC-GSH cycle, the action of glutathione transferases (GST), and their subsequent storage in vacuoles [78].

## 5. Conclusions

There are not many examples in which the possible role of melatonin as a natural safener has been studied. However, studies carried out clearly show the excellent possibilities of melatonin in reducing the damage caused by the herbicide in the crop, activating anti-oxidative defenses, reducing oxidative stress, and presenting a greater tolerance to the herbicide. That is, melatonin behaves as a usual safener, but with greater protective skills and without the prejudice of the use of synthetic substances in addition to the herbicide, thus being a clear biological bet. In the case of the fungicides tested, very positive results have been observed, verifying not only the individual and joint efficacy of melatonin and fungicide in the control of plant disease, but also finding a synergistic action between both, which allows a lower dosage of the artificial fungicide, which is always preferable for using ecological pest control. The analyzed studies provide evidence for the adequate role of melatonin as safener and can serve as a proposal for its possible applications in agricultural and biotechnological areas.

However, there are many studies to be carried out in this context, such as the following: (i) given the breadth of herbicides used, studies should be performed in categories, verifying the role as a safener of melatonin in each case; (ii) provide quantitative data on the capacity of melatonin to reduce crop damage; (iii) possibilities of decrease in the applied dose of herbicides, with the consequent environmental benefit; (iv) improvement in the rate of detoxification of the herbicide, destination, and toxicity of the by-products; (v) study the great synergistic possibilities between melatonin and fungicides as a possible eco-friendly tool in the fight of fungal pests; (vi) extend the studies to other pesticides, such as insecticides, etc.

Finally, we should not overlook the possible effects of melatonin treatments on crops. Although melatonin is a safe molecule in humans (no intoxication or serious side effects have been reported), its excessive presence in treated crops could have unintended consequences. Several aspects of the application of melatonin in agriculture have been previously discussed, analyzing its pros and cons and giving a series of tips to stimulate research in the field of health and the environment related to melatonin [40,79]. In brief, melatonin (or natural phytomelatonin-rich extracts) may be effective as a plant protector and biostimulant in crops, inducing immune and tolerance responses, activating primary and secondary metabolism, and redox and hormonal networks. It can be used in root and leafy treatments and is a cheap chemical substance. Some possible problems or cons could be the need for more studies on persistence and ecotoxicity and their hormonal roles in animals, among others.

## Figures and Tables

**Table 1:** Some safeners used in different crops.

Crop	Safener	Application Mode
Maize		
	Naphthalic anhydride	Seed treatment
	Dichlormid	Pre-emergence
	Benoxacor	Pre-emergence
	Furilazole	Pre-emergence
	Isoxadifen-ethyl	Post-emergence
	Cyprosulfamide	Pre- and post-emergence
	AD67, MG191	Pre-emergence
Sorghum		
	Cyometrinil	Seed treatment
	Oxabetrinil	Seed treatment
	Flurazole	Seed treatment
	Fluxofenim	Seed treatment
Grasses		
	Cloquintocet-mexyl	Post-emergence
	Fenchlorazole-ethyl	Post-emergence
	Mefenpyr-diethyl	Post-emergence
Rice		
	Daimuron	Water surface
	Cumyluron	Water surface
	Dimepiperate	Water surface
	Fenclorim	Pre-emergence
	Isoxadifen-ethyl	Post-emergence
Cotton	Dietholate	Seed treatment
Soybean	Triapenthenol	Pre-emergence

**Table 2:** Studies with different pesticides to verify the possible safener activity of melatonin in plants.

Pesticide	Common Name	Plant	Year	Effects	Reference
Herbicide	Butafenacil	Rice	2013	High tolerance to herbicide	[67]
Herbicide	Paraquat	Pea	2017	High photosynthesis	[68]
		Poplar	2018	High tolerance to stress and low damage	[69]
Herbicide	Bentazone	Batata	2020	High growth and tolerance to herbicide Low damage	[70]
Fungicide	Carbendazim	Tomato	2019	Low damage, high stress tolerance, and fungicide metabolizing	[71]
Fungicide	Ethylicin	Tobacco	2018	Synergistic action, suppression of virulence, low fungicide doses, and eco-friendly alternative	[72]
Fungicide	Infinito	Potato	1993	Synergistic action, low dosage, and high efficacy	[73]
